# A Rare Cause of Fatty Liver and Elevated Aminotransferase Levels: Chanarin-Dorfman Syndrome: A Case Report

**DOI:** 10.4061/2011/341372

**Published:** 2011-01-20

**Authors:** Özdal Ersoy, Canan Alkım, Mehmet Derya Onuk, Hüseyin Demirsoy, Dilek Argon

**Affiliations:** ^1^Department of Gastroenterology, Şişli Etfal Training and Research Hospital, 34377 Istanbul, Turkey; ^2^Department of Hematology, Şişli Etfal Training and Research Hospital, 34377 Istanbul, Turkey

## Abstract

Chanarin-Dorfman syndrome is a rare, inherited metabolic disorder of neutral lipid storage characterized by ichthyosis, lipid vacuoles in leukocytes, and involvement of several internal organs, mostly the liver. Since the initial case was reported by Dorfman in 1974, nearly 50 cases have been reported, and the majority were from Middle East countries. Here, we report a 20-year-old patient with ichthyosis from Turkey, diagnosed as Chanarin-Dorfman syndrome presented with asypmtomatic elevated transaminases and hepatosteatosis, and also briefly review the updated clinical implications and management of this rarely seen syndrome. Prompt diagnosis of this syndrome avoids further unnecessary investigations in patients with ichthyosis

## 1. Introduction

Chanarin-Dorfman syndrome (CDS) is a rare, inherited disorder of lipid metabolism and is also known as a multisystem (including liver, eyes, ears, skeletal muscle, and central nervous system) neutral lipid storage disease with sine qua non ichthyosis due to an acylglycerol recycling defect. It is inherited as an autosomal recessive pattern and its incidence is unknown and mutations in ABHD5/CGI58 gene are associated with CDS and because of these mutations, triacylglycerols accumulate in cytosolic droplets in multiple organs. Here, we report a rare cause of fatty liver due to this neutral lipid storage disorder in an asymptomatic 20-year-old Turkish woman born to consanguineous parents, presenting with ichthyosis and referred from dermatology department for further investigation of the elevated transaminases. Although the clinical progression in patients with CDS ranges from mild to fatal, our case has mild clinical symptoms and she is the oldest case diagnosed as CDS to date. Awareness of this rare condition, but diagnosed with simple peripheral blood examination, helps in prompt diagnosis and avoids further investigations in the differentiation of fatty liver in patients with ichthyosis. Nearly a total of 50 patients with CDS have been reported worldwide till 2010, but further evaluation of patients with ichthyosis, bearing in mind the possibility of CDS, may increase the number of reported CDS cases in the literature, and the pathogenesis and progression of the disease will become clearer in the future. Briefly, here we reviewed the main updated clinicopathological findings of CDS in the light of another new adult patient with CDS from Turkey who needs to be added to the list of CDS cases reported in the literature.

## 2. Case Report

**Figure 1:**
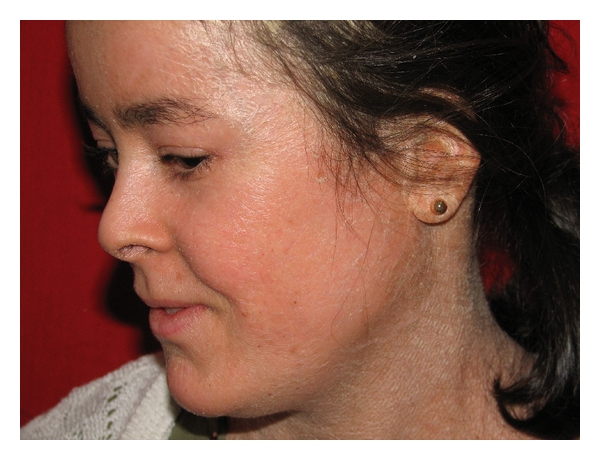
White scaly, dry, and firm face of the patient, mostly prominent in the ear and the skin under the hair.

**Figure 2:**
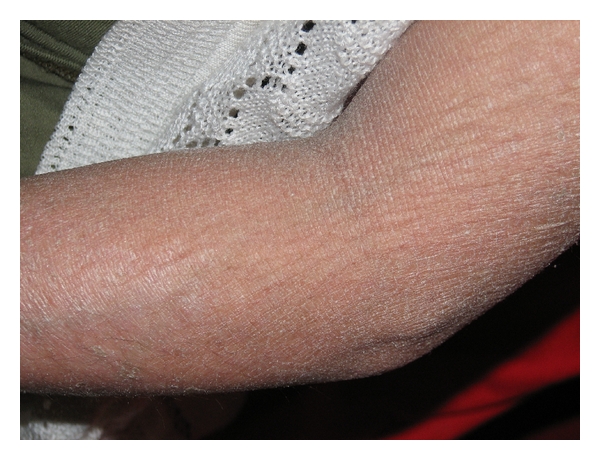
Dry skin of the forearm. Scaling and dryness are more pronounced in the skin of the extremity than the skin of her face.

**Figure 3:**
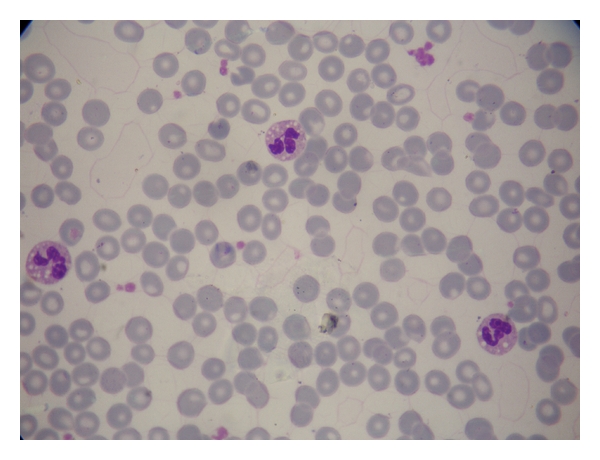
Blood smear showing multiple lipid vacuoles in the cytoplasms of three neutrophils.

A 20-year-old woman admitted to dermatology department with scaly skin particularly involving her face, arms, and hands. The punch biopsy of the skin revealed a lamellar ichthyosis. She had asymptomatic elevated aminotransferase levels. The patient had scaly and erythematous skin present since birth and her parents also had noticed that she had frequent diaper rash, however she was only given symptomatic treatment provided in the form of local ointments. There was no history of difficulty in feeding, icterus, dark-colored urine, or clay-colored stools. She had also unilateral congenital cataract and strabismus but her vision was normal. While growing up, her skin continued to be diffusely hyperkeratotic and scaly. In the last two years, the patient started to complain about muscle cramps and pain which her parents ignored. When her skin got worse during winter, she was brought to the dermatology clinic where the transaminases were firstly found elevated. Dermatologic examination revealed fine white translucent scaly skin lesions in face, abdomen, and arms (Figures 1 and 2). The palms, soles, teeth, and nails were normal. There was a generalised abdominal distension with a firm, nontender hepatomegaly, and the liver was palpable 1 cm below the costal margin in the midclavicular line. Spleen was not palpable. Growth and development was normal but according to her parents she was a little mentally retarded, although her IQ level was not ever calculated. There was no neurological deficit, and muscle tone, power, and deep tendon reflexes were all normal. Gait was normal. Ophthalmological examination revealed a corticonuclear cataract and strabismus and mild ectropion. Audological examination was normal. The rest of the systemic examination revealed no pathological findings. Aspartate aminotransferase (AST) and alanine aminotransferase (ALT), creatinine kinase (CK) and lactate dehydrogenase (LDH) levels were all increased (AST: 61 U/L, ALT:59 U/L normal for both: 0–40 U/L; CK:765 U/L normal: 25–160; LDH:893 U/L, normal: <225). Complete blood count, erytrocyte sedimentation rate, fasting blood sugar, renal (including creatinine and glomerular filtration rate) and thyroid function tests (thyroid stimulating hormone and free T4), alkaline phosphatase, gamma-glutamyl transpeptidase, bilirubin, albumin, and seruloplasmin levels and coagulation parameters were within normal ranges. Protein electrophoresis, immunoglobulin levels, and blood lipid profile were normal. Viral hepatitis markers (anti-HAV total, HBsAg, anti-HBs, anti-HBc IgG, anti-HCV, HCV-RNA, and HBV-DNA) were all negative. Bile acid levels could not be measured. Anti-mitochondrial antibody (AMA), antinuclear antibody (ANA) and anti-liver-kidney microsomes (LKM-1) were negative where antismooth muscle antibody (ASMA) was positive in 1/40 dilution. Hematological parameters including serum iron, total iron binding capacity, and ferritin were normal. Due to increased levels of muscle enzymes (CK and LDH) and mild msucle pains; electromyography (EMG) was performed and the EMG findings were normal. Ultrasound of the abdomen revealed mild hepatomegaly with increased echotexture suggesting grade 2 hepatosteatosis. Lipid droplets were seen on Giemsa-stained peripheral blood smear as vacuoles in granulocytes and monocytes (Figure 3). Finally performed needle liver biopsy showed degenaration in the liver parenchymal cells with inflammatory infiltrate and moderate macrovesicular steatosis and large lipid vacuoles in the cytoplasms of the hepatocytes.

A diagnosis of CDS was made on dermatological, peripheral blood smear and liver biopsy findings. The patient was put on a low-fat diet in consultation with a dietician, and treatment was given only to dermatological disorders. She was on followup from the gastroenterology outpatient clinic for 2 years after the diagnosis was made and she was asymptomatic apart from the ichthyosiform condition and alternating ALT and AST and CK levels during the followup controls. Afterwards the patient quitted her followup and the contact with the patient is lost.

## 3. Discussion

Chanarin-Dorfman syndrome is a rare, inherited (autosomal recessive) disorder of lipid metabolism characterized by ichthyosis, lipid vacuoles in leukocytes, and involvement of several internal organs which was first described by Dorfman et al. [1] and Chanarin et al. [2]. Since its first description, nearly 45 cases showing all the characteristics of ichthyosis and Chanarin-Dorfman syndrome were reported in the literature. Although European and Asian patients have been reported, the majority of cases were mostly from the Mediterranean and Middle East countries, especially from Turkey and in consanguineous matings [3–11]. Our case was also born to a consanguineous family, and she is from the center Anatolian region of Turkey.

CDS is a multisystem triglyceride storage disease due to a lysosomal inborn error of neutral lipids, and clinically it is an autosomal recessive form of nonbullous congenital ichthyosiform erythroderma (NCIE) demonstrating fine white scaling on an erythematous background. It is characterized by the presence of congenital ichthyosis with deposition of lipid droplets in multiple organs. Specific defects have been postulated to be an abnormal recycling of triglyceride-derived mono- or diacylglycerol to specific phospholipids [12] or to be a defect in the catabolism of long-chain fatty acids [13].

 Babies with CDS may be born with a collodion membrane and may present bilateral ectropion and eclabion. Hair, nails, teeth and mucosa are normal; however, abnormal pattern of hair distribution may be seen. In addition to NCIE, affected individuals have other organ involvement, the most frequent of which is hepatomegaly (described in nearly 50% of the reported cases) and hepatosteatosis. These two main clinical features of CDS; NCIE and hepatomegaly or liver steatosis, are present in all patients but with differing severities as rarely steatohepatitis and cirrhosis [11, 14, 15]. Variability in dermatological and hepatical severity is not fully understood but, it must be kept in mind that the rapid progression as steatohepatitis has been found to occur in CDS. The case described here has congenital ichthyosis and hepatomegaly with moderately elevated transaminases and hepatosteatosis proven by ultrasound and liver biopsy. Muscle weakness, ataxia, neurosensory hearing loss, eye findings (subcapsular cataracts, nystagmus, and strabismus), microcephaly, cardiomyopathy, and mental retardation may also be present but the involvement of these other organs and systems is much more variable; for example, muscle involvement is demonstrated in 69%, developmental delay or mental retardation in 35%, and cataracts in 46% of the subjects with neutral lipid storage disease [10]. In this presented case, hepatosteatosis, mild mental retardation (not confirmed with IQ measurement), subcapsular cataract, mild ectropion, strabismus, and raised levels of serum muscle enzyme were present, which is very similar to another Turkish patient recently published by Durdu et al. [16].

In CDS, histologically, intracellular lipid droplets are found in most tissues such as in dermis, nerve cells, hepatocytes, sweat gland cells. In the liver biopsy of our patient, large lipid vacuoles were seen in the hepatocytes.

A diagnosis of CDS is easily confirmed by a simple blood smear. Characteristic lipid droplets are observed in the cytoplasm of granulocytes which are described as Jordan's anomaly [17], and this is a pathagnomonic sign for CDS. Since CDS may present with just skin findings and because of the wide spectrum of clinical variability, mild cases might be easily underdiagnosed. Thus, CDS should be investigated for the differential diagnosis of all the congenital ichthyoses. Therefore it is recommended that every case of ichtyosis should have an analysis of a fresh peripheral blood smear examined by a hematologist/pathologist at a local clinical laboratory with special attention to detect lipid-containing vacuoles in circulating leukocytes. Our case, who is nearly asymptomatic except the scaly skin and mildly elevated transaminases and moderate hepatomegaly, demonstrates the value of such a screening blood smear examination showing these vacuoles in her neutrophils which led us to the correct diagnosis. Various previous work have emphasized the importance of peripheral blood smear examination of clinically unaffected members for Jordans' anomaly to help in the detection of heterozygous carriers [18]. Alternatively, lipid stains (oil red O, Sudan III) can be used to detect cytoplasmic lipid droplets in keratinocytes and fibroblasts using fresh frozen skin sections. Durdu et al. also demonstrated vacuoles in the keratinocytes by cytologic examination of the Tzanck smear test, which was not reported previously [16].

In 2001, in 9 families from the Mediterranean basin segregating triglyceride storage disease with impaired long-chain fatty acid oxidation, Lefevre et al. identified mutations in the human comparative gene identification-58 (CGI-58) gene which are linked to CDS [19]. Recently, Yamaguchi focused on the functions and protein interactions of CGI-58 on the surface of lipid droplets in the regulation of fat mobilization in cells [20]. Other genes have been identified for lipid metabolism disorders in which ichthyosis is part of a syndrome that involves other organs such as liver, CNS, eyes, and ears, but these disorders include the classic form of X-linked ichthyosis, like Refsum disease where intracellular lipid (phytanic-acid cholesterol esters) inclusions can also be seen [21]; however, refsum disease was excluded by the absence of ataxia and retinitis pigmentosa in our patient.

Still the physiologic roles of the CGI-58 protein remain to be clarified, the sensitivity of the mutation analysis of this gene is expected to be high in individuals with CDS. Although we could not perform any genetic test in our patient, the indications for direct DNA testing are confirmation of the clinical diagnosis, distinguishing CDS from other forms of icthyosis, genetic counseling and recurrence risk calculation, and prenatal diagnosis for couples with unknown mutations. Besides genetic testing, screening of cultured fibroblasts for triglyceride accumulation could also be performed for the confirmation of the diagnosis.

There is no effective treatment of CDS, but a low-fat diet is reported to decrease skin and liver findings; still, there is no documented evidence that dietary modifications alter the course of the disease. Retinoids can be useful in the treatment of skin and muscle manifestations but cannot be given to patients having deranged liver functions [18]. In our case we only recommended a diet low in long-chain fatty acids and kept her on followup every 6 months in order to monitor hepatic damage. The transaminase levels showed alternating high-and-low pattern as seen in hepatosteatosis but other liver function tests were nonprogressive for the 2 years after the diagnosis. Afterwards the patient quitted her followup and the contact with the patient is lost.

As a brief, here we reviewed the main updated clinicopathological findings of CDS in the light of another new adult patient with CDS from Turkey who needs to be added to the list of CDS cases reported to date. Simple, easy, and inexpensive peripheral blood smear may provide important information in a patient with ichthyosis to make an early diagnosis of CDS and can avoid unnecessary further tests for prompt diagnosis of CDS. Early assessment of the liver functions should also be performed for the possible liver involvement in this syndrome in order to follow up and give treatment when needed.
